# Isolation and identification of compounds from *Kalanchoe pinnata* having human alphaherpesvirus and vaccinia virus antiviral activity

**DOI:** 10.1080/13880209.2017.1310907

**Published:** 2017-04-11

**Authors:** Matthew Cryer, Kyle Lane, Mary Greer, Rex Cates, Scott Burt, Merritt Andrus, Jiping Zou, Paul Rogers, Marc D. H. Hansen, Jillybeth Burgado, Panayampalli Subbian Satheshkumar, Craig W. Day, Donald F. Smee, F. Brent Johnson

**Affiliations:** a Department of Microbiology and Molecular Biology, Brigham Young University, Provo, UT, USA;; b Department of Biology, Brigham Young University, Provo, UT, USA;; c Department of Chemistry and Biochemistry, Brigham Young University, Provo, UT, USA;; d Department of Nutrition, Dietetics and Food Science, Brigham Young University, Provo, UT, USA;; e Department of Physiology and Developmental Biology, Brigham Young University, Provo, UT, USA;; f Poxvirus and Rabies Branch, Centers for Disease Control and Prevention, Atlanta, GA, USA;; g Institute for Antiviral Research, Utah State University, Logan, UT, USA

**Keywords:** HHV, VACV, antiviral, cytotoxicity

## Abstract

**Context:** *Kalanchoe pinnata* (Lam.) Pers. (Crassulaceae) is a succulent plant that is known for its traditional antivirus and antibacterial usage.

**Objective:** This work examines two compounds identified from the *K. pinnata* plant for their antivirus activity against human alphaherpesvirus (HHV) 1 and 2 and vaccinia virus (VACV).

**Materials and methods:** Compounds KPB-100 and KPB-200 were isolated using HPLC and were identified using NMR and MS. Both compounds were tested in plaque reduction assay of HHV-2 wild type (WT) and VACV. Both compounds were then tested in virus spread inhibition and virus yield reduction (VYR) assays of VACV. KPB-100 was further tested in viral cytopathic effect (CPE) inhibition assay of HHV-2 TK-mutant and VYR assay of HHV-1 WT.

**Results:** KPB-100 and KPB-200 inhibited HHV-2 at IC_50_ values of 2.5 and 2.9 μg/mL, respectively, and VACV at IC_50_ values of 3.1 and 7.4 μg/mL, respectively, in plaque reduction assays. In virus spread inhibition assay of VACV KPB-100 and KPB-200 yielded IC_50_ values of 1.63 and 13.2 μg/mL, respectively, and KPB-100 showed a nearly 2-log reduction in virus in VYR assay of VACV at 20 μg/mL. Finally, KPB-100 inhibited HHV-2 TK- at an IC_50_ value of 4.5 μg/mL in CPE inhibition assay and HHV-1 at an IC_90_ of 3.0 μg/mL in VYR assay.

**Discussion and conclusion:** Both compounds are promising targets for synthetic optimization and *in vivo* study. KPB-100 in particular showed strong inhibition of all viruses tested.

## Introduction

Human alphaherpesvirus (HHV) 1 and 2 (commonly known as herpes simplex viruses types 1 and 2) are DNA viruses responsible for a number of infections including orolabial and genital lesions, localized and disseminated infection in neonates, encephalitis, gingivostomatitis, keratoconjunctivitis, herpes gladitorum, herpes whitlow and treatment-resistant infection of immunocompromised persons. As of 2012, an estimated 3.7 billion and 417 million people worldwide, between the ages of 15 and 49 years, were infected with HHV-1 and HHV-2, respectively (Looker et al. 2015a,b). The drug acyclovir (ACV) is the most commonly used treatment for both primary and recurrent HHV infections, including prophylactic treatment to mitigate recurrent infections and, in the case of the ACV prodrug valacyclovir, to prevent asymptomatic transmission. A long-term developing issue surrounding the use of ACV is the appearance of drug-resistant virus mutants. ACV-resistant HHV is rare (0.27%) in immunocompetent adults, but in a study in the Netherlands ACV-resistant isolates were much more common (7–14%) in immunocompromised adults (Stránská et al. 2005). It is generally recognized that ACV-resistance is highly prevalent in HIV-infected patients.

Following drug entry into cells, ACV is tri-phosphorylated and then blocks the activity of virus-specific DNA polymerase. Phosphorylation to the monophosphate level, beginning the phosphorylation chain, is usually carried out by the virus-specific enzyme thymidine kinase (TK). If this enzyme is deficient or mutated to inactivity, the virus becomes ACV resistant. Reports indicate approximately 95% of acyclovir-resistant HHV strains carry a mutant TK gene (Pottage & Kessler 1995; Stránská et al. 2004). TK-negative and TK-low producer mutants are also resistant to other ACV derivatives such as penciclovir (Gilbert et al. 2002). Viral mutations in the UL23 gene are responsible for the TK-negative or TK-low producer status of these ACV-resistant strains (Coen & Schaffer 1980). Foscarnet and cidofovir are second-line treatments for ACV-resistant, or nucleoside analog-resistant HHV strains, and are also used to treat other herpes viruses such as human cytomegalovirus (CMV) (Oberg 1989; Castelo-Soccio et al. 2010). However, these polymerase inhibitors have low bioavailability that limits their usage to intravenous administration and they are highly nephrotoxic (Deray et al. 1989; Izzedine et al. 2005). It is important to find anti-herpes drugs that inhibit ACV-resistant clinical strains.

Vaccinia virus (VACV) is a member of the Poxviridae family and is closely related to variola virus (VARV), the causative agent of smallpox (Moss 2013). VACV is a useful proxy for *in vitro* and *in vivo* study when evaluating therapeutic potential of prospective VARV treatments (CDER 2007). Classically, VACV strains have been used successfully as live smallpox vaccines to eradicate VARV infections. They are largely innocuous except in cases of host immune deficiency. In some immune deficient patients, symptoms of generalized Vaccinia including skin lesions occur (James et al. 2006). Complications of smallpox vaccination also included accidental autoinfection, spread to casual contacts, generalized Vaccinia, eczema vaccinatum, postvaccinal encephalopathy, roseola Vaccinia and progressive Vaccinia (Lane et al. 1969; Bray & Wright 2003).

Smallpox is a category A biological threat and concerns exist that it could be used as a weapon of mass destruction. The Institute of Medicine (Institute of Medicine (US) Committee on the Assessment of Future Scientific Needs for Live Variola Virus 1999) recommended that at least two antiviral drugs be developed and stockpiled to defend against the threat of VARV being used as a weapon of bioterrorism (LeDuc et al. 2002). Tecovirimat, a small molecule with activity against orthopoxviruses, is the safest and most effective treatment of VARV that has been validated to date (Mucker et al. 2013). It is important to find additional anti-pox therapeutic agents that could be used in the event of smallpox outbreaks and complications following the possible re-introduction of live VACV vaccines to the public.

Extracts, fractions and identified compounds from *Kalanchoe pinnata* have previously shown activity against a number of DNA and RNA viruses, including HHV-1 and HHV-2 (Supratman et al. 2001; Greer et al. 2010; Aoki et al. 2014). Traditionally, *K. pinnata* has been used as a medicinal plant to treat a range of diseases including bacterial infections, cancer and inflammation throughout areas where the plant grows natively.

In this report, we describe our studies on the anti-HHV-2 and anti-VACV activities of two compounds, KPB-100 and KPB-200, obtained from the roots of *K. pinnata* (Lam.) Pers. (Crassulaceae). Both compounds were found to be inhibitors of both viruses. Additionally, we describe the anti-HHV-1 activity of KPB-100.

## Materials and methods

### Plant tissue collection and extraction

The plant tissue collection and organic chemical extraction procedure of *K. pinnata* roots have been previously described (Greer et al. 2010). In the current study, components in the extracts were subjected to further separation and purification by HPLC.

### Isolation and identification

*High-performance liquid chromatography (HPLC): K. pinnata* extracts were analyzed on an Agilent 1100 HPLC system, and the fractions were separated and collected by Agilent 1200 fraction collector (model G1364C). Analytical columns Phenomenex Luna (2) (4.6 × 150 mm 3 um) were used for the separation with mobile phase acetonitrile and water. The UV detector at wavelength of 254 nm was used.

*Mass Spectrometry (MS)*: The MS data for KPB-100 were collected on an Orbitrap LC-MS in ion-mode with electrospray ionization (ESI). The sample was prepared using a small amount of the NMR sample (acetone-d6 solvent) dissolved in a solution of 99.9% acetonitrile, 0.1% formic acid. The MS data for KPB-200 were collected on an Agilent MSD time-of-flight instrument with ESI. The sample was prepared using a small amount of the NMR sample (D_2_O solvent) dissolved in H_2_O to back-exchange the deuterated alcohols with hydrogen.

*Nuclear magnetic resonance spectroscopy (NMR)*: NMR data were collected on a Varian NMR-S 500 MHz console using a Varian broadband probe with pulsed-field gradients and a sample temperature of 294 K. Chemical shifts are reported in delta (*δ*) units, in parts per million (ppm) downfield from the reference compound. Compound KPB-200 was dissolved in D_2_O with a reference compound of 4,4-dimethyl-4-silapentane-1-sulfonic acid (DSS). Compound KPB-100 was dissolved in acetone-d6 with a reference compound of tetramethylsilane (TMS). The concentration of KPB-100 was too low to obtain a directly acquired carbon-13 spectrum (^13 ^C NMR), so the ^13 ^C chemical shifts were obtained from indirectly acquired 2D NMR spectra. Final structures were determined using 2D NMR.

### Plaque reduction assays

*Standard plaque reduction assay to determine anti-HHV-2 WT activity*: Testing of KPB-100 and KPB-200 for antiviral activity was performed against HHV-2 WT as previously described (Greer et al. 2010). KPB-100 and KPB-200 were diluted in Dulbecco’s Modified Eagle’s Medium (DMEM) supplemented with 0.11% sodium bicarbonate, 5% Cosmic calf serum (Hyclone), 10 mM HEPES buffer and 50 μg/mL gentamicin. Cell monolayers of C1008 cells in 24-well culture plates (Corning) were exposed to drug at varying concentrations, infected with HHV-2, strain G (ATCC VR-734), at a multiplicity of infection (MOI) 0.001, incubated at 36 °C, then processed by fixation and immunoperoxidase staining (Luker et al. 1991). The plaques were observed microscopically and counted. Virus controls were included in addition to drug toxicity controls. End points of drug activity were expressed as 50% inhibitory concentration (IC_50_) values and were calculated by regression analysis.

*Viral CPE inhibition assay to determine KPB-100 anti-HHV-2 TK-mutant activity*: HHV-2 thymidine kinase mutant (TK-minus; TK-), CI-10161 was a patient isolate obtained on 11 November 1986 from Bruce Ronnie at Burrows Welcome. It was amplified one time in MA-104 cells. HHV-2 TK- CI-1253 (18 February 1991) was a patient isolate obtained on 05 February 1992 from Bruce Ronnie at the department of medicine at the University of British Colombia and amplified one time in Vero cells. HHV-2 (E194) was the standard wild-type E194 strain from the USU archives. KPB-100 was suspended in water (100 μg/mL). It was diluted 1/2 in minimum essential medium (MEM) solution, then diluted down further in half-log dilutions in MEM solution with 50 μg/mL gentamicin and 2% foetal bovine serum. Each dilution was added to 5 wells of a 96-well plate with an overnight monolayer of 80–100% confluent Vero-76 cells. Acyclovir and cidofovir were tested in parallel as controls. Three wells of each dilution were then infected with virus, and two wells remained uninfected as toxicity controls. The MOI was approximated 0.001 for CI-1253, 0.002 for CI-10161 and 0.017 for E194. When untreated virus control wells reached near complete cytopathic effect (CPE), which was day-four post-infection in each case, plates were stained with neutral red dye (which measures cell viability, thus can be used for quantifying the vitality of both virus-infected and uninfected, drug-treated cell cultures) for approximately 2 h, then supernatant dye was removed and the incorporated dye was extracted in 50:50 Sorensen citrate buffer/ethanol, then read on a spectrophotometer at 540 nm. Optical density (OD) values were normalized based on cell and virus controls, then IC_50_, 50% cytotoxic concentration (CC_50_) values, and Selectivity Index (SI) were calculated by regression analysis.

*Plaque reduction assay to determine anti-VACV activity*: KPB-100 and KPB-200 were suspended in water (100 μg/mL). Vero-76 cells were seeded to 24-well plates for KPB-100 and 12-well plates for KPB-200 and grown overnight to near confluence. Each well was then inoculated with a 1/3200 dilution of VACV (WR), obtained from ATCC, for KPB-100 and a 1/1000 dilution of the virus for KPB-200 and incubated for 1 hour to allow for infection. KPB-100 and KPB-200 were then diluted in MEM solution containing 2% FBS to 20, 6.3, 2.0, 0.063, 0.2, 0.63, 0.02 and 20, 10, 5, 2.5, 1.25, 0.63, 0.31, 0.156 μg/mL, respectively, and each dilution added to duplicate virus-infected wells. An identical plate was run in parallel without virus to test for cytotoxicity of KPB-100 but was not run for KPB-200 due to limited material availability. Plates were incubated at 37 °C with 5% CO_2_ for 3 days, and then plaques were counted under a microscope. Small satellite plaques that emerged after day two of incubation were excluded. Cidofovir was tested in parallel as a positive control. KPB-100 toxicity plates were stained with neutral red dye for approximately 2 h, then supernatant dye was removed and the incorporated dye was extracted in 50:50 Sorensen citrate buffer/ethanol, then read on a spectrophotometer, and OD values were compared to untreated control wells. KPB-200 toxicity was estimated by visual inspection of infected wells in areas of the cells where viral plaques were not present. OD values were normalized based on cell and virus controls; the IC_50_ and CC_50_ were calculated by regression analysis.

### HHV-1 VYR assay

A viral CPE inhibition assay was first performed using a similar methodology as earlier HHV-2 TK-mutant assays. Vero-76 cells were infected with a 1/50 dilution of HHV-1 (v2424). KPB-100 was suspended in water at 100 μg/mL and diluted in 2× MEM solution containing 4% FBS at varying concentrations. Supernatant fluid was then collected from each compound concentration and tested for virus titre using a standard endpoint dilution of 50% cell culture infectious dose (CCID_50_) assay in quadruplicate wells. Titre calculations were done using the Reed–Muench (Reed & Muencha 1948) equation. The concentration of compound required to reduce virus yield by 1 log_10_ or 90% (IC_90_ value) was calculated by regression analysis. Generally, the IC_90_ value obtained by VYR assay approximates an IC_50_ value obtained by either plaque reduction or CPE inhibition assay. IC_50_ values for VYR (twofold reduction in virus titre) are usually not reported, because this is insignificant reduction in virus yield and does not result in protection of cells from virus-induced CPE.

### VACV spread inhibition assay

HeLa cells were seeded into 96-well plates. After 24 h, cells were infected with VACV WR-GFP virus (VV.NP-S-EGFP). GFP expressed from the viral genome is targeted to nucleus with nuclear localization signal (Earl et al. 2003; Johnson et al. 2008). KPB-100 and KPB-200 were then diluted in MEM solution to 16, 4, 1, 0.25 μg/mL. Plates were incubated at 37 °C for 24 h. Cells were fixed with 4% formaldehyde and stained with DAPI (binds to DNA). Cytosine arabinoside (cytarabine, 46 μg/mL) was used as a positive control to inhibit virus spread. When cytarabine is used, no new virus is produced after the initial GFP positive cells, which is 0% spread. In the absence of inhibitor, virus spread will cause secondary infection and hence increase in GFP positive cells (100% spread). Cells were scanned and analyzed using ArrayScan High Content Platform as previously described (Johnson et al. 2008). IC_50_ was calculated using per cent spread relative to cytarabine control, where 0% spread is defined as 100% inhibition.

### VACV VYR assay

HeLa cells seeded into 48-well plates were infected with VACV WR-GFP. The cells were washed 1 h post-infection and harvested (for input) or incubated in the absence and the presence of KPB-100 and KPB-200 (20, 10, 5, 2.5 and 1.25 μg/mL) for 24 h at 37 °C. Cells were harvested, lysed by three freeze-thaw cycles and virus yield was determined by plaque assay in BS-C-40 cells. Plaques were stained by crystal violet and quantitated 48 h after infection. The increase in virus yield was calculated by subtracting the 24 h titre with input viral titre. The concentration of compound required to reduce virus yield by 1 log_10_ or 90% (IC_90_ value) was calculated by regression analysis.

## Results

### Mass spectrometry

*KPB-100* The base peak was 529.2055 (M–Na^+^) with a prominent peak at 545.1793 (M–K^+^) which both yield a high-res *m*/*z* of 506.2156; which produces an empirical formula of C_26_H_34_O_10_.

*KPB-200* The base peak was 484.1624 (M–H^+^) with a prominent peak at 984.3432 (2 M–NH_4_^+^) which both yield a high-res *m*/*z* of 483.1531.

### Nuclear magnetic resonance spectroscopy

Proton nuclear magnetic resonance (^1 ^H NMR) data are reported as follows: s (singlet), d (doublet), dd (doublet of doublet), ddd (doublet of doublet of doublet), m (multiplet). Coupling constants are reported in Hertz (Hz).

*KPB-100* ^1 ^H NMR (500 MHz, D_2_O) δ 6.91 (d, *J* = 1.9 Hz, 1 H), 6.78 (d, *J* = 7.9 Hz, 1 H), 6.69 (s, 1 H), 6.63 (dd, *J* = 7.9,1.9 Hz, 1 H), 6.25 (s, 1 H), 4.15 (d, *J* = 7.2 Hz, 1 H), 4.10 (d, *J* = 10.6 Hz, 1 H), 4.03 (dd, *J* = 9.6, 2.4 Hz, 1 H), 3.81 (s, 3 H), 3.81 (d, *J* = 11.2 Hz, 1 H), 3.78 (s, 3 H), 3.74 (d, *J* = 4.2 Hz, 2 H), 3.50 (s, 3 H), 3.50 (m, 1 H), 3.36 (dd, *J* = 8.6, 8.6 Hz, 1 H), 3.26 (dd, *J* = 8.5, 7.3 Hz, 1 H), 3.20 (dd, *J* = 9.6, 3.1 Hz, 1 H), 3.17 (dd, *J* = 11.2, 10.0 Hz, 1 H), 2.91 (dd, *J =* 15.9, 11.5 Hz, 1 H), 2.81 (dd, *J* = 15.9, 4.5 Hz, 1 H), 2.02 (m, 1 H), 1.92 (m, 1 H)

^13^C NMR (125 MHz, D_2_O) δ 147.8, 147.6, 147.4, 145.0, 137.3, 132.6, 129.5, 121.9, 115.0, 114.0, 113.4, 111.8, 104.5, 76.7, 73.6, 70.0, 67.8, 65.5, 63.7, 55.6, 55.4, 55.2, 46.8, 44.7, 38.4, 32.9

*KPB-200* ^1^H NMR (500 MHz, (CD_3_)_2_CO) δ 6.10 (m, 1 H), 6.02 (s, 2 H), 6.00 (m, 1 H), 5.99 (m, 1 H), 5.08 (d, *J* = 7.7 Hz, 1 H), 4.74 (dd, *J* = 11.9, 2.5 Hz, 1 H), 4.46 (dd, *J* = 11.9, 9.6 Hz, 1 H), 3.94 (ddd, *J* = 9.4, 9.4, 2.5 Hz, 1 H), 3.78 (s, 6 H), 3.65 (dd, *J* = 9.0, 9.0 Hz, 1 H), 3.59 (dd, *J* = 9.0, 7.6 Hz, 1 H), 3.52 (dd, *J* = 9.4, 9.4 Hz, 1 H), 3.48 (s, 3 H)

^13^C NMR (125 MHz, (CD_3_)_2_CO) δ 170.7, 163.6, 160.7, 160.5, 150.3, 143.8, 121.9, 110.1, 101.8, 99.8, 98.3, 97.3, 78.5, 76.4, 75.6, 73.7, 66.7, 58.9, 57.9

### Compound structures

See Figure 1.

**Figure 1.**
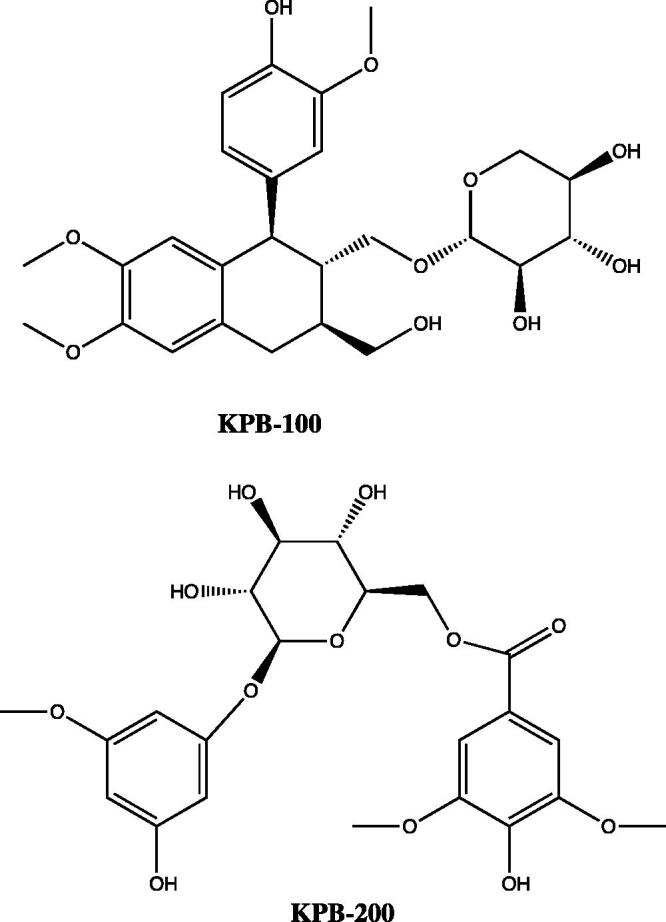
Compound structures.

### Standard plaque reduction assay against HHV-2 WT

KPB-100 and KPB-200 were evaluated for their HHV-2 WT activity in a plaque reduction assay where they yielded IC_50_ values of 2.5 and 2.9 μg/mL, respectively.

### Viral CPE inhibition assay against HHV-2 TK-

KPB-100, acyclovir and cidofovir were evaluated for their activity against HHV-2 TK- in a viral CPE inhibition assay where they yielded mean IC_50_ values of 4.55, 12.2 and 0.71 μg/mL, respectively (Table 1). In the same series against HHV-2 WT, KPB-100, acyclovir and cidofovir yielded IC_50_ values of 5.4, 0.49 and 4.3 μg/mL, respectively.

**Table 1.** Anti-HHV-2 activity of compounds from *K. pinnata* roots in plaque reduction assays.

	Antiviral activity (μg/ml)			
Compound	Virus	IC_50_^a^	CC_50_^b^	SI^c^
KPB-100	HHV-2 E194	5.4	>25	>4.6
Acyclovir	HHV-2 E194	0.49	>100	>204
Cidofovir	HHV-2 E194	4.3	>100	>23
KPB-100	HHV-2 TK- 1253	4.6	>25	>5.4
Acyclovir	HHV-2 TK- 1253	9.3	>100	>11
Cidofovir	HHV-2 TK- 1253	0.22	>100	>450
KPB-100	HHV-2 TK- 10161	4.5	>25	>5.6
Acyclovir	HHV-2 TK- 10161	15	>100	>6.7
Cidofovir	HHV-2 TK- 10161	1.2	>100	>83

^a^ IC_50_ = 50% inhibitory concentration.

^b^ CC_50_ = 50% cytotoxic concentration of compound in uninfected cultures.

c SI = Selectivity Index CC_50_/IC_50_.

### VYR assay against HHV-1

KPB-100 was evaluated for its activity against HHV-1 WT in a VYR assay where it yielded an IC_90_ value of 3.0 μg/mL.

### Plaque reduction assay against VACV

KPB-100, KPB-200 and cidofovir were evaluated for their activity against VACV in a plaque reduction assay where they yielded IC_50_ values of 3.1, 7.4 and 9.7 (mean) μg/mL, respectively. No toxicity at the 50% level was observed for either compound up to 20 μg/mL (as measured by neutral red uptake into cells), but 20% toxicity was estimated visually with 20 μg/mL of KPB-100, while no toxicity was seen with the diluent control (20% water in MEM).

### VACV spread inhibition assay

In a VACV spread inhibition assay, KPB-100 and KPB-200 yielded IC_50_ values of 1.63 and 13.19 μg/mL, respectively. At 16 μg/mL of both the compounds yielded negative spread relative to cytarabine of −98.5 and −63.2, respectively (Table 2).

**Table 2.** Anti-VACV activity of compounds from *K. pinnata* roots in virus spread inhibition assay.

% Spread compared to cytarabine control				
	Drug Concentration (μg/ml)			
Compound	16	4	1	0.25
KPB-100	−98.5	0.29	119.3	128.7
KPB-200	−63.2	80.0	112.5	119.9

### VACV VYR assay

**Figure 2.**
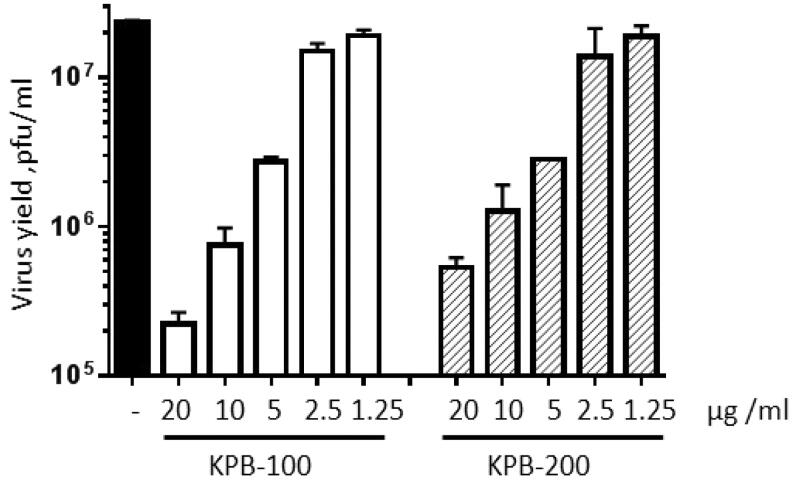
VACV VYR assay. Units for virus yield are plaque forming units per millilitre (pfu/mL). Concentration of compounds in μg/mL.

In a VACV yield inhibition assay, KPB-100 at a concentration of 20 μg/mL had a nearly two-log reduction in virus titre compared to control (Figure 2). While both compounds were found to be active, similar to plaque reduction assay, KPB-100 exhibited higher inhibition than KPB-200.

## Discussion

Crude extracts and isolated compounds from *K. pinnata* leaves, aerial parts and roots have previously shown activity against a variety of viruses including HHV-1, HHV-2, Epstein-Barr and hepatitis C (Supratman et al. 2001; Greer et al. 2010; Aoki et al. 2014). *Kalanchoe pinnata* has been used as a traditional medicinal plant to treat a range of diseases including bacterial infections, cancer, and inflammation, and the *Kalanchoe* genus generally has been the subject of a number of *in vitro* and *in vivo* studies. Identified compounds with antivirus activity from *K. pinnata* include flavonoids, phenols and bufadienolides (Supratman et al. 2001; Aoki et al. 2014). This paper reported for the first time the anti-VACV activity for any compound or fraction from *K. pinnata*, and the anti-HHV activity of scaphopetalone, KPB-100 as well.

In plaque reduction assays KPB-100 and KPB-200 were shown to be low μg/mL inhibitors of HHV-2 WT. Upon further testing, KPB-100 was approximately as potent against TK- strains as it was against the WT(E194) strain, whereas acyclovir potency was markedly lower in TK- isolates compared to the WT. Cidofovir showed greater potency against the TK- isolates than against the WT HHV. KPB-100 was also a potent inhibitor of HHV-1 in VYR assay. Further *in vitro* testing will assess the spectrum of utility of KPB-100 against TK-negative and Tk-low producer strains, as well as studying KPB-200 against TK-negative strains.

KPB-100 and KPB-200 were assessed for their activity against VACV in plaque reduction assays with cidofovir as a positive control. All compounds tested were potent at low μg/mL concentrations, with KPB-100 being the strongest inhibitor. Similarly, in virus yield inhibition assay, both KPB-100 and KPB-200 exhibited anti-VACV activities.

Both KPB-100 and KPB-200 achieved profound negative per cent spread in a VACV spread inhibition assay at 16 μg/mL, as defined by GFP expression relative to the positive control, cytarabine. Additionally, KPB-100 showed a high degree of potency at nearly 100% inhibition of virus spread at 4 μg/mL. Further studies are required to establish the mechanism of action of these compounds and to determine inhibitory step(s) in the VACV life cycle.

The cytotoxicity of KPB-100 and KPB-200 was <20% cytopathic effect at 25 μg/mL as measured by CPE in plaque reduction. However, because of limited material availability, CC_50_ and CC_100_ data points have not yet been found for either compound. Near-term studies will focus on characterizing cytotoxic effects *in vitro* and toxic and pharmacokinetic effects *in vivo*.

## Conclusions

Both tested compounds proved to be potent inhibitors of HHV-2 and VACV. Additionally, KPB-100 showed strong activity of acyclovir-resistant HHV-2 TK-mutant isolates and HHV-1.

The carbon structures of KPB-100 and KPB-200 are high value targets for future synthetic optimization, and KPB-100 may be directly useful as an antiviral therapeutic agent. Treatment areas of highest priority include treatment-sensitive and treatment-resistant HHV-1 and HHV-2 via topical or systemic administration, smallpox and progressive Vaccinia. Further antivirus spectrum of activity tests may yield additional indications in the future.

## References

[CIT0001] Aoki C, Hartati S, Santi MR, Lydwina Firdaus R, Hanafi M, Kardono LBS, Yohko S, Pratiwi S, Hak H. 2014. Isolation and identification of substances with anti-hepatitis C cirus activities from *Kalanchoe pinnata*. Int J Pharm Pharm Sci. 6:211–215.

[CIT0002] Bray M, Wright ME. 2003. Progressive *Vaccinia*. Clin Infect Dis. 36:766–774. 12627361 10.1086/374244

[CIT0003] Castelo-Soccio L, Bernardin R, Stern J, Goldstein SA, Kovarik C. 2010. Successful treatment of acyclovir-resistant *Herpes simplex* virus with intralesional cidofovir. Arch Dermatol. 146:124–126. 20157021 10.1001/archdermatol.2009.363 PMC2874880

[CIT0004] Food and Drug Administration Center for Drug Evaluation and Research (CDER). 2007. Draft guidance for industry on smallpox (*Variola*) infection: developing drugs for treatment or prevention; availability. Docket No. 2007D-0439.

[CIT0005] Coen DM, Schaffer PA. 1980. Two distinct loci confer resistance to acycloguanosine in *Herpes simplex* virus type 1. Proc Natl Acad Sci USA. 77:2265–2269. 6246531 10.1073/pnas.77.4.2265 PMC348694

[CIT0006] Deray G, Martinez F, Katlama C, Levaltier B, Beaufils H, Danis M, Rozenheim M, Baumelou A, Dohin E, Gentilini M, et al. 1989. Foscarnet nephrotoxicity: mechanism, incidence and prevention. Am J Nephrol. 9:316–321. 2554731 10.1159/000167987

[CIT0007] Earl PL, Americo JL, Moss B. 2003. Development and use of a *Vaccinia* virus neutralization assay based on flow cytometric detection of green fluorescent protein. J Virol. 77:10684–10688. 12970455 10.1128/JVI.77.19.10684-10688.2003 PMC228521

[CIT0008] Gilbert C, Bestman-Smith J, Boivin G. 2002. Resistance of herpesviruses to antiviral drugs: clinical impacts and molecular mechanisms. Drug Resist Updat. 5:88–114. 12135584 10.1016/s1368-7646(02)00021-3

[CIT0009] Greer MR, Cates RG, Johnson FB, Lamnaouer D, Ohai L. 2010. Activity of acetone and methanol extracts from thirty-one medicinal plant species against *Herpes simplex* virus types 1 and 2. Pharm Biol. 48:1031–1037. 20731556 10.3109/13880200903468873

[CIT0010] Institute of Medicine (US) Committee on the Assessment of Future Scientific Needs for Live Variola Virus. 1999. Assessment of future scientific needs for live variola virus. Washington DC: National Academies Press. 25101435

[CIT0011] Izzedine H, Launay-Vacher V, Deray G. 2005. Antiviral drug-induced nephrotoxicity. Am J Kidney Dis. 45:804–817. 15861345 10.1053/j.ajkd.2005.02.010

[CIT0012] James W, Berger T, Elston D. 2006. Andrews' diseases of the skin: clinical dermatology. London, UK: Saunders Elsevier.

[CIT0013] Johnson MC, Damon IK, Karem KL. 2008. A rapid, high-throughput *Vaccinia* virus neutralization assay for testing smallpox vaccine efficacy based on detection of green fluorescent protein. J Virol Methods. 150:14–20. 18387679 10.1016/j.jviromet.2008.02.009

[CIT0014] Lane JM, Ruben FL, Neff JM, Millar JD. 1969. Complications of smallpox vaccination, 1968. N Engl J Med. 281:1201–1208. 4186802 10.1056/NEJM196911272812201

[CIT0015] LeDuc JW, Damon I, Relman DA, Huggins J, Jahrling PB. 2002. Smallpox research activities: US interagency collaboration, 2001. Emerg Infect Dis. 8:743–745. 12095449 10.3201/eid0807.020032 PMC2730338

[CIT0016] Looker KJ, Magaret AS, May MT, Turner KM, Vickerman P, Gottlieb SL, Newman LM. 2015a. Global and regional estimates of prevalent and incident herpes simplex virus type 1 infections in 2012. PLoS One. 10:e0140765. 26510007 10.1371/journal.pone.0140765 PMC4624804

[CIT0017] Looker KJ, Magaret AS, Turner KM, Vickerman P, Gottlieb SL, Newman LM. 2015b. An estimate of the global prevalence and incidence of herpes simplex virus type 2 infection. PLoS One. 10:e114989. 25608026 10.1371/journal.pone.0114989 PMC4301914

[CIT0018] Luker G, Chow C, Richards DF, Johnson FB. 1991. Suitability of infection of cells in suspension for detection of herpes simplex virus. J Clin Microbiol. 29:1554–1557. 1653270 10.1128/jcm.29.7.1554-1557.1991 PMC270157

[CIT0019] Moss B. 2013. Poxviridae. In: Knipe DM, Howley PM, editor. Fields virology. Vol. 2. Philadelphia, PA: Lippincott Williams & Wilkins; p. 2130.

[CIT0020] Mucker EM, Goff AJ, Shamblin JD, Grosenbach DW, Damon IK, Mehal JM, Holman RC, Carroll D, Gallardo N, Olson VA, et al. 2013. Efficacy of tecovirimat (ST-246) in nonhuman primates infected with variola virus (Smallpox). Antimicrob Agents Chemother. 57:6246–6253. 24100494 10.1128/AAC.00977-13 PMC3837858

[CIT0021] Oberg B. 1989. Antiviral effects of phosphonoformate (PFA, foscarnet sodium). Pharmacol Ther. 40:213–285. 2543994 10.1016/0163-7258(89)90097-1

[CIT0022] Pottage JC, Jr., Kessler HA. 1995. Herpes simplex virus resistance to acyclovir: clinical relevance. Infect Agents Dis. 4:115–124. 8548189

[CIT0023] Reed LJ, Muencha H. 1938. Simple method of estimating fifty per cent endpoints. Am J Epidemiol. 27:493–497.

[CIT0024] Stránská R, Schuurman R, Nienhuis E, Goedegebuure IW, Polman M, Weel JF, Wertheim-Van Dillen PM, Berkhout RJ, van Loon AM. 2005. Survey of acyclovir-resistant herpes simplex virus in the Netherlands: Prevalence and characterization. J Clin Virol. 32:7–18. 15572000 10.1016/j.jcv.2004.04.002

[CIT0025] Stránská R, van Loon AM, Polman M, Beersma MF, Bredius RG, Lankester AC, Meijer E, Schuurman R. 2004. Genotypic and phenotypic characterization of acyclovir-resistant herpes simplex viruses isolated from haematopoietic stem cell transplant recipients. Antivir Ther (Lond.). 9:565–575. 15456088

[CIT0026] Supratman U, Fujita T, Akiyama K, Hayashi H, Murakami A, Sakai H, Koshimizu K, Ohigashi H. 2001. Anti-tumor promoting activity of bufadienolides from *Kalanchoe pinnata* and *K. daigremontiana* x *tubiflora*. Biosci Biotechnol Biochem. 65:947–949. 11388478 10.1271/bbb.65.947

