# Corrigendum

**DOI:** 10.1080/13880209.2017.1322793

**Published:** 2017-05-03

Matthew C, Kyle L, Mary G, Rex C, Scott B, Merritt A, Jiping Z, Paul R, Marc DHH, Jillybeth B, et al. 2017. Isolation and identification of compounds from *Kalanchoe pinnata* having human alphaherpesvirus and vaccinia virus antiviral activity. Pharmaceutical Biology. 55:1586–1591.

https://doi.org/10.1080/13880209.2017.1310907

When the above article was first published online, there were mistakes in the author’s name which have now been corrected in both online and print version.

